# High frequency of isavuconazole use over voriconazole for chronic pulmonary aspergillosis: a retrospective observational study on tolerability and therapeutic drug monitoring

**DOI:** 10.1128/aac.00892-26

**Published:** 2026-09-16

**Authors:** Federico Fama, Claudia Conflitti, Aurora Civati, Dario Cattaneo, Alessandro Torre, Maria Vittoria Cossu, Silvia Borghetti, Debora Visigalli, Stefania Vimercati, Spinello Antinori, Agostino Riva, Andrea Gori, Marta Colaneri, Marco Schiuma

**Affiliations:** 1 Department of Infectious Diseases, Luigi Sacco Hospital, Regional Center for Infectious Diseases (CEREMI), Lombardy Region 665657 , Milan, Italy; 2 Centre for Multidisciplinary Action in Global Health (MAGH), Department of Biomedical and Clinical Sciences (DIBIC), Luigi Sacco Hospital, University of Milan, Milan, Italy; 3 National PhD Programme in One Health Approaches to Infectious Diseases and Life Science Research, Department of Public Health, Experimental and Forensic Medicine, University of Pavia 19001, Pavia, Italy; 4 Department of Biomedical Sciences, Humanitas University, Pieve Emanuele, Milan, Italy; 5 IRCCS Humanitas Research Hospital, Rozzano, Milan, Italy; 6 Pharmacy Unit, ASST Fatebenefratelli-Sacco, Milan, Italy; University of Iowa, Iowa City, lowa, USA

**Keywords:** chronic pulmonary aspergillosis, isavuconazole, voriconazole, therapeutic drug monitoring

## Abstract

Chronic pulmonary aspergillosis (CPA) is a progressive fungal infection associated with significant morbidity and mortality. Voriconazole, a standard first-line treatment, is limited by poor tolerability, unpredictable pharmacokinetics, and extensive drug-drug interactions. Isavuconazole, a newer triazole with a more favorable safety profile, is currently recommended only as later-line therapy due to limited evidence in CPA. To evaluate the rate of switch from voriconazole to isavuconazole, and to assess the tolerability, pharmacokinetic profile, and drug costs of isavuconazole in a real-world CPA cohort. Single-center retrospective observational study of 40 patients with CPA enrolled at Luigi Sacco University Hospital, Milan, between October 2020 and October 2024, with therapeutic drug monitoring (TDM) throughout treatment. Of 26 patients initiated on voriconazole, 53.8% required a switch to isavuconazole, primarily due to failure to achieve therapeutic plasma concentrations (71.4%) and adverse events. Overall, 70% (28/40) of the cohort ultimately received isavuconazole. Among 233 isavuconazole TDM measurements, 17.6% fell outside the therapeutic range (1–5 mg/L), and 50% of patients required at least one dose adjustment. No patient discontinued isavuconazole due to adverse events, and no treatment failure or relapse occurred among isavuconazole-treated patients. TDM-guided dose reduction to 100 mg daily in 39.3% of patients yielded an estimated drug cost saving of 21.9%. Isavuconazole demonstrated a favorable tolerability profile with no treatment discontinuations due to adverse events. Proactive TDM proved clinically valuable in guiding dose optimization and reducing drug costs, supporting isavuconazole as a viable alternative for long-term CPA management.

## INTRODUCTION

Chronic pulmonary aspergillosis (CPA) is one of the main clinical manifestations of *Aspergillus* spp.—a leading cause of severe pulmonary disease with high morbidity and mortality—developing typically in patients with pre-existing structural lung damage or immunocompromising conditions (1, 2). According to Denning, CPA is estimated to have a global annual incidence of 1,837,272 cases (1). Recent data from a systematic review and meta-analysis of 70 studies estimate a pooled 5-year mortality of 32% (95% CI: 25–39%), with considerable variability depending on underlying comorbidities and CPA subtype (3).

The management of CPA is challenging primarily due to the poor tolerability and limited manageability of the available antifungal agents, compounded by comorbidities and potential drug interactions rather than by a lack of intrinsically effective therapeutic options.

The preferred treatment consists of oral triazoles, with outpatient oral therapy being generally recommended. Voriconazole or itraconazole are considered first-line options, while posaconazole is an alternative with similar efficacy but variable cost, depending on generic availability across different healthcare settings (2, 4).

However, this class of antifungals is associated with potentially severe side effects, such as drug-induced liver damage (DILI) and QTc prolongation, a narrow therapeutic range requiring frequent plasma level monitoring (TDM), and significant drug interactions, which increase the risk of toxicity or treatment failure if therapeutic levels are not maintained.

Isavuconazole is a newer triazole, approved in 2015 for the treatment of invasive aspergillosis and mucormycosis. Compared to other azoles, it offers several advantages such as: (i) lower hepatic toxicity; (ii) QTc shortening rather than prolongation; and (iii) fewer drug-drug interactions (5).

Although sporadic data support its use as a primary therapeutic option, the limited evidence on the efficacy and safety profile of isavuconazole positions it as a later-line treatment for CPA (6).

The aim of this study is to evaluate the manageability of isavuconazole in a real-world setting, in terms of the rate of switch from voriconazole to isavuconazole, as well as its tolerability and pharmacokinetic profile in patients with CPA followed at Luigi Sacco University Hospital.

## MATERIALS AND METHODS

### Study design

We conducted a single-center, retrospective observational study based on data collected in a routine clinical setting. Patients evaluated for antifungal therapy for CPA referred to Luigi Sacco University Hospital in Milan between October 2020 and October 2024 were included. For eligible patients, clinical and therapeutic follow-up information available at the time of data extraction was collected through May 2026. Medical records of the participants were pseudo-anonymized and abstracted on standardized data collection forms.

### Study participants

We included patients aged >18 years referred to the clinic with a diagnosis of CPA defined according to the ESCMID/ERS consensus as:

1. Compatible appearance in thoracic imaging, including cavitation (with or without fungal ball), pericavitary infiltrates, pleural thickening, nodules, consolidation, or progressive parenchymal fibrosis.
2. Direct evidence of *Aspergillus* infection or an immunological response to *Aspergillus* spp. (e.g., a Galactomannan antigen test in bronchoalveolar lavage [BAL] or blood, a culture exam positive for *Aspergillus* species in sputum or BAL, *Aspergillus fumigatus* IgG or *Aspergillus* IgG Western blot positivity).
3. Exclusion of other diseases.
4. Disease presence for at least 3 months, verified through retrospective review of clinical records and imaging (2, 4).

Exclusion criteria were underage patients, pregnant women, and patients who were administered antifungal treatment other than voriconazole or isavuconazole.

### Patient characteristics

The following data were systematically collected for each patient:

- Demographic and anthropometric data: age, sex, country of origin, ethnicity, height, weight, and BMI.
- Clinical data: radiological findings, antifungal regimen (drug choice and posology), adverse events (including DILI, QTc prolongation, and phosphenes), and treatment outcome (cure, relapse, and death).
- Comorbidities: cardiovascular diseases, hypertension, chronic lung disease (chronic pulmonary obstructive disease [COPD], asthma, emphysema, and bronchiectasis), chronic kidney disease, liver disease, rheumatological disease, diabetes, and neurological/psychiatric conditions, HIV infection, history of solid organ or bone marrow transplant, active or recent malignancy (defined as a cancer diagnosis or completion of anticancer therapy within the 12 months preceding antifungal initiation).
- *Aspergillus* infection-specific risk factors: history of thoracic surgery, prior tuberculosis or non-tuberculous mycobacterial infection, lung cavitation, hypoalbuminemia, and corticosteroid use (inhaled or systemic).
- Microbiological data: *Aspergillus* serology (*Aspergillus* IgG Western blot, *A. fumigatus* IgG and IgE), galactomannan antigen (blood, sputum, or BAL), and microscopy and culture of sputum and/or BAL.
- Laboratory data: TDM of voriconazole (therapeutic range 1.5–5 mg/L) and isavuconazole (1–5 mg/L).

The isavuconazole therapeutic range of 1–5 mg/L is the reference range adopted by our institutional laboratory, informed by available real-world evidence and consensus expert guidance (7, 8). This range is broadly consistent with the 1–4 mg/L efficacy target and >4.8 mg/L toxicity threshold recently proposed by the working group of the Italian Society of Clinical Biochemistry and Clinical Molecular Biology (SIBioC) and with the 1–5 mg/L range adopted in other real-world settings (9, 10).

### Outcomes

The primary outcome of the study was to evaluate the rate of switch from voriconazole to isavuconazole. Switch was defined as a composite outcome, driven by any of the following events, at the discretion of the treating physician: onset of clinical adverse events (phosphenes/photophobia, hallucinations, neuropathy, or skin rash), abnormal laboratory findings (AST or ALT > 5 × ULN in asymptomatic patients, or >3 × ULN with total bilirubin > 2 × ULN in symptomatic patients, or total bilirubin > 3 × ULN regardless of symptoms), electrolyte imbalance, significant drug interactions arising from the introduction of a new concomitant therapy, and/or persistent failure to achieve target plasma concentrations.

Secondary outcomes evaluated: (i) isavuconazole tolerability; (ii) isavuconazole pharmacokinetic profile during long-term treatment through TDM, including the proportion of patients requiring dose adjustment; (iii) treatment outcomes (cure, relapse, and death); and (iv) drug acquisition costs associated with TDM-guided dose optimization.

Treatment success (cure) was defined as clinical improvement/stabilization of symptoms and radiological response (absence of progression or reduction of CPA-related lesions on thoracic imaging). Treatment failure was defined as clinical, radiological, or microbiological worsening despite at least 6 months of antifungal therapy at therapeutic plasma concentrations, or the need to switch to an alternative antifungal agent due to lack of efficacy. Relapse was defined as recurrence of clinical and/or radiological signs of active CPA after documented treatment success occurring during follow-up after treatment completion. Outcome definitions were based on routine clinical assessment and retrospective review of medical records.

Drug costs were estimated from the hospital pharmacy perspective using the institutional acquisition cost of isavuconazole (€34.8 per 100 mg capsule). Total costs were calculated for each patient based on actual treatment duration and prescribed dose and compared against a hypothetical scenario assuming standard dosing (200 mg daily) throughout. Only direct drug acquisition costs were included; costs associated with therapeutic drug monitoring (e.g., laboratory analyses and outpatient visits), adverse event management, and other indirect costs were not considered.

### Study methodology

Plasma samples for the proactive TDM of isavuconazole and/or voriconazole were collected during routine outpatient evaluations, starting from day 7 after the initiation of treatment. Specifically, trough level samples were taken 12 h after the last dose of voriconazole and 24 h after the last dose of isavuconazole. Sampling was repeated monthly, with more frequent monitoring in cases where the therapeutic window was not achieved. Patients underwent regular outpatient follow-ups at least every 4 weeks during antifungal therapy. Dose adjustments were made at the discretion of the treating physician based on TDM results and/or clinical assessment. Plasma concentrations of antifungal agents were quantified at the Pharmacy Unit laboratory of ASST Fatebenefratelli-Sacco using a commercially available CE-IVD liquid chromatography–tandem mass spectrometry (LC-MS/MS) kit (MassTox TDM Series, Chromsystems Instruments & Chemicals GmbH, Gräfelfing, Germany).

### Statistics

The collected data were described in terms of mean and standard deviation (or median and IQR, if appropriate) for continuous variables and in terms of counts and percentages for categorical variables. Differences between groups were assessed with the chi-square test (or Fisher’s exact test, as appropriate) for categorical variables, and the Mann–Whitney test for continuous variables. Differences were considered statistically significant for *P* values < 0.05.

## RESULTS

**Fig 1:**
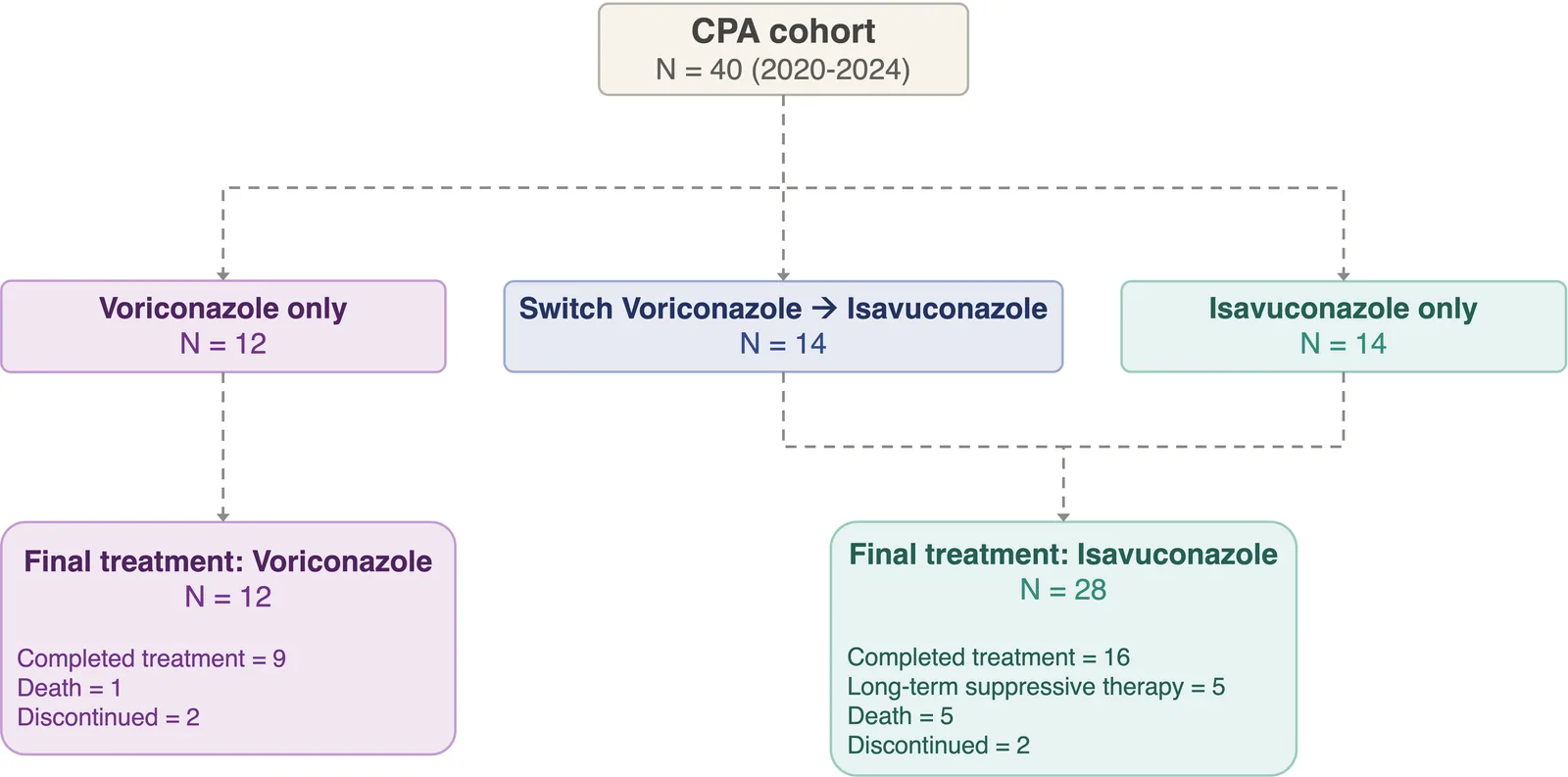
Patient flow through initial antifungal allocation, switch from voriconazole to isavuconazole, and final treatment outcomes.

The study population consisted of 40 patients diagnosed with CPA. At the start of therapy, 26 patients (65%) were prescribed voriconazole, while 14 (35%) received isavuconazole; of the 26 patients who started voriconazole, 14 (53.8%) subsequently switched to isavuconazole (Fig. 1). The median age of the total population was 68 years (IQR: 56.8–74.0); patients starting isavuconazole tended to be older than those starting voriconazole (70.5 vs 63.5 years, *P* = 0.054). Among comorbidities, chronic lung diseases were the most prevalent comorbidities (32/40, 80.0%), with COPD or asthma affecting 55.0% (22/40) of the cohort, followed by bronchiectasis (11/40, 27.5%) and emphysema (10/40, 25.0%). The isavuconazole group had more individuals with immunocompromising illnesses (64.3% vs 30.8%, *P* = 0.087), in particular, active or recent diagnosis of cancer (57.1% vs 23.1%; *P* = 0.071). Further details are reported in Table 1.

**TABLE 1:** Baseline demographic and clinical characteristics of the study population stratified by initial antifungal treatment^*a*^

	Starting therapy		Total (*N* = 40)
	Voriconazole (***N*** = 26)	Isavuconazole (*N* = 14)	
Demographics			
Gender (M)	12 (46.2)	10 (71.4)	22 (55.0)
Age (median [IQR])	63.5 [54.5–72.5]	70.5 [67.5–77.0]	68 [56.8–74.0]
BMI (median [IQR])	22.9 [20.0–24.2]	21 [18.2–24.4]	22 [19.5–24.3]
Smoking habit			
Former smoker	12 (46.2)	9 (64.3)	21 (52.5)
Active smoker	5 (19.2)	4 (28.6)	9 (22.5)
Non-smoker	9 (34.6)	1 (7.1)	10 (25.0)
Comorbidity count (median [IQR])	3 [2.0–4.8]	3 [2.2–4.8]	3 [2–5]
Cardiovascular diseases	5 (19.2)	3 (21.4)	8 (20.0)
Chronic lung disease	19 (73.1)	13 (92.9)	32 (80.0)
COPD/asthma	12 (46.2)	10 (71.4)	22 (55.0)
Emphysema	6 (23.1)	4 (28.6)	10 (25.0)
Bronchiectasis	6 (23.1)	5 (35.7)	11 (27.5)
Diabetes	7 (26.9)	1 (7.1)	8 (20.0)
Liver diseases	3 (11.5)	3 (21.4)	6 (15.0)
Rheumatological diseases	3 (11.5)	1 (7.1)	4 (10.0)
Chronic kidney disease	2 (7.7)	0 (0.0)	2 (5.0)
Immunocompromising conditions	8 (30.8)	9 (64.3)	17 (42.5)
Active or recent solid cancer	6 (23.1)	8 (57.1)	14 (35.0)
Lung tumor	1 (3.8)	4 (28.6)	5 (12.5)
HIV infection	3 (11.5)	1 (7.1)	4 (10.0)
Steroids for systemic use	3 (11.5)	1 (7.1)	4 (10.0)
Solid organ transplant	1 (3.8)	1 (7.1)	2 (5.0)
*Aspergillus* infection risk factors			
Steroids for inhalation	8 (30.8)	5 (35.7)	13 (32.5)
Hypoalbuminemia	5 (19.2)	7 (50.0)	12 (30.0)
History of lung cavitation	6 (23.1)	4 (28.6)	10 (25.0)
Post-TB	3 (11.5)	2 (14.3)	5 (12.5)
Post-NTM	2 (7.7)	2 (14.3)	4 (10.0)
Other	1 (3.8)	0 (0.0)	1 (2.5)
History of NTM infection	3 (11.5)	4 (28.6)	7 (17.5)
History of TB infection	4 (15.4)	3 (21.4)	7 (17.5)
History of thoracic surgery	1 (3.8)	3 (21.4)	4 (10.0)

^*a*^ COPD, Chronic obstructive pulmonary disease; TB, tuberculosis; NTM, non-tuberculous mycobacteria.

Radiological, serological, and microbiological findings are summarized in Table 2. Cavitation and nodules were the most common radiological features (50.0% each), and *Aspergillus fumigatus* was the predominant species identified (64.5%).

**TABLE 2:** Radiological findings, serological tests, and microbiological results in 40 patients with chronic pulmonary aspergillosis^*a*^

Finding/test	Positive (no.)	Positive (%)
Radiology		
Cavitation	20/40	50.0%
Nodules	20/40	50.0%
Consolidation	12/40	30.0%
Parenchymal fibrosis	11/40	27.5%
Mediastinal lymphadenopathy	4/40	10.0%
Fungus ball	3/40	7.5%
Tree-in-bud	2/40	5.0%
Serology		
*Aspergillus* IgG Western Blot	25/32	78.1%
*A. fumigatus* IgG	19/27	70.4%
Serum galactomannan	6/36	16.7%
Microbiology		
BAL galactomannan	27/32	84.4%
BAL culture	18/31	58.1%
Sputum culture	13/19	68.4%
Species identified		
*A. fumigatus*	20/31	64.5%
*A. niger*	5/31	16.1%
*A. terreus*	2/31	6.5%
*A. flavus*	1/31	3.2%
Other/unspecified	3/31	9.7%

^*a*^ Denominators vary according to test availability. BAL, bronchoalveolar lavage.

### Pharmacology

**Fig 2:**
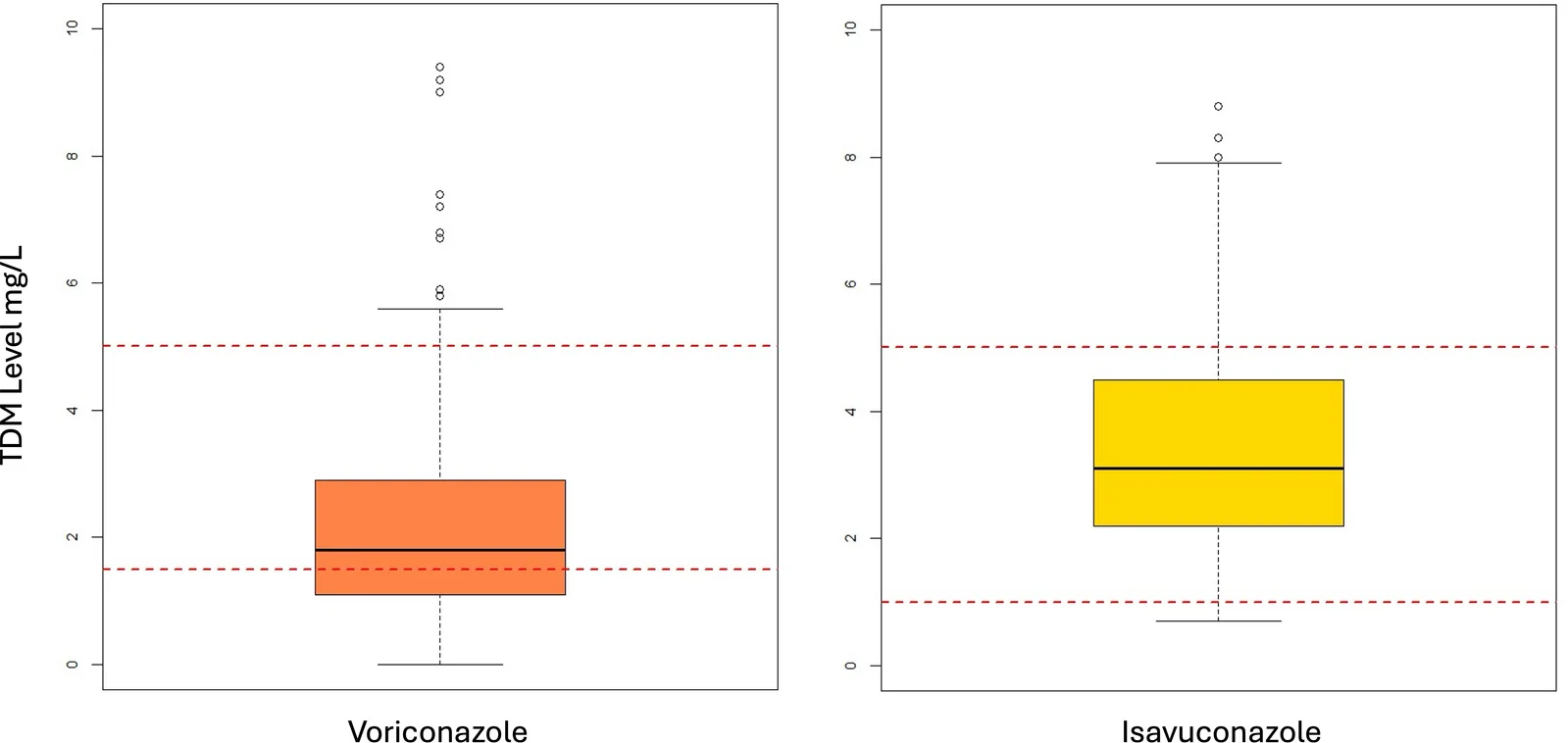
Distribution of all therapeutic drug monitoring (TDM) measurements for voriconazole (left) and isavuconazole (right); the dotted red lines represent the therapeutic range thresholds (1.5–5 mg/L for voriconazole, 1–5 mg/L for isavuconazole).

Overall, 435 TDM measurements were performed across the study population: 202 for voriconazole and 233 for isavuconazole (Fig. 2). The median initial TDM value was 3.4 mg/L (IQR: 1.5–5.1) for voriconazole and 5.1 mg/L (IQR: 3.3–6.2) for isavuconazole.

#### Voriconazole

Among the 26 patients who initiated treatment with voriconazole, 14 (53.8%) required a switch to isavuconazole, while 12 (46.2%) remained on the initial drug throughout. Patients who received only voriconazole had a median treatment duration of 7.0 months (IQR: 4.7–9.9), and therapeutic plasma levels were achieved in 113/202 TDM measurements (55.9%).

The need for dosage adjustments was higher in patients receiving voriconazole, with 80.8% requiring at least one posology change, compared to 50.0% (14/28) of those on isavuconazole (*P* = 0.166). Overall, patients required a median of 2 posology changes (IQR: 2–3) before switching to isavuconazole or completing therapy. Patients who switched to isavuconazole had significantly lower initial voriconazole trough levels compared to those who did not switch (median 3.0 mg/L [IQR: 1.2–3.8] vs 4.5 mg/L [IQR: 3.0–7.6]; *P* = 0.0327).

Among the 14 patients who switched from voriconazole to isavuconazole, failure to achieve adequate plasma concentrations was the most frequent reason, occurring in 10/14 patients (71.4%).

The switch occurred after a mean of 3.2 months (median 2.9 months, IQR: 1.9–4.3) and of the 11 patients in the switch group who required dose adjustment, 4 (36.4%) underwent three or more posology changes before transitioning to isavuconazole. Additional or concomitant reasons for switching included: palliative intent (2/14, 14.3%), patient preference (2/14, 14.3%), treatment failure (2/14, 14.3%), cardiovascular adverse events (including QTc prolongation) (2/14, 14.3%), and single cases (7.1% each) of ophthalmic adverse effects (including phosphenes), drug-drug interactions (with antimycobacterial drugs), gastrointestinal intolerance, and hyperkalemia. The median overall treatment duration (from initiation of voriconazole to end of isavuconazole) was 17.4 months (IQR: 11.0–24.2).

#### Isavuconazole

##### Rationale and extent of isavuconazole use

Among patients started on isavuconazole as the primary regimen, the main driver was its more favorable drug-drug interaction profile (10/14, 71.4%), driven by concurrent antimycobacterial (35.7%) or other interacting medications (35.7%), followed by two patients with baseline hepatopathy (14.3%, one patient with HCV-related cirrhosis and one with markedly elevated transaminases [ALT 220 U/L] while receiving chemotherapy for non-small-cell lung adenocarcinoma), and single cases of previous azole failure (7.1%) and prior azole-induced QTc prolongation (7.1%). The 14 patients treated exclusively with isavuconazole had a median treatment duration of 8.7 months (IQR: 5.4–17.7).

##### Therapeutic drug monitoring

**Fig 3:**
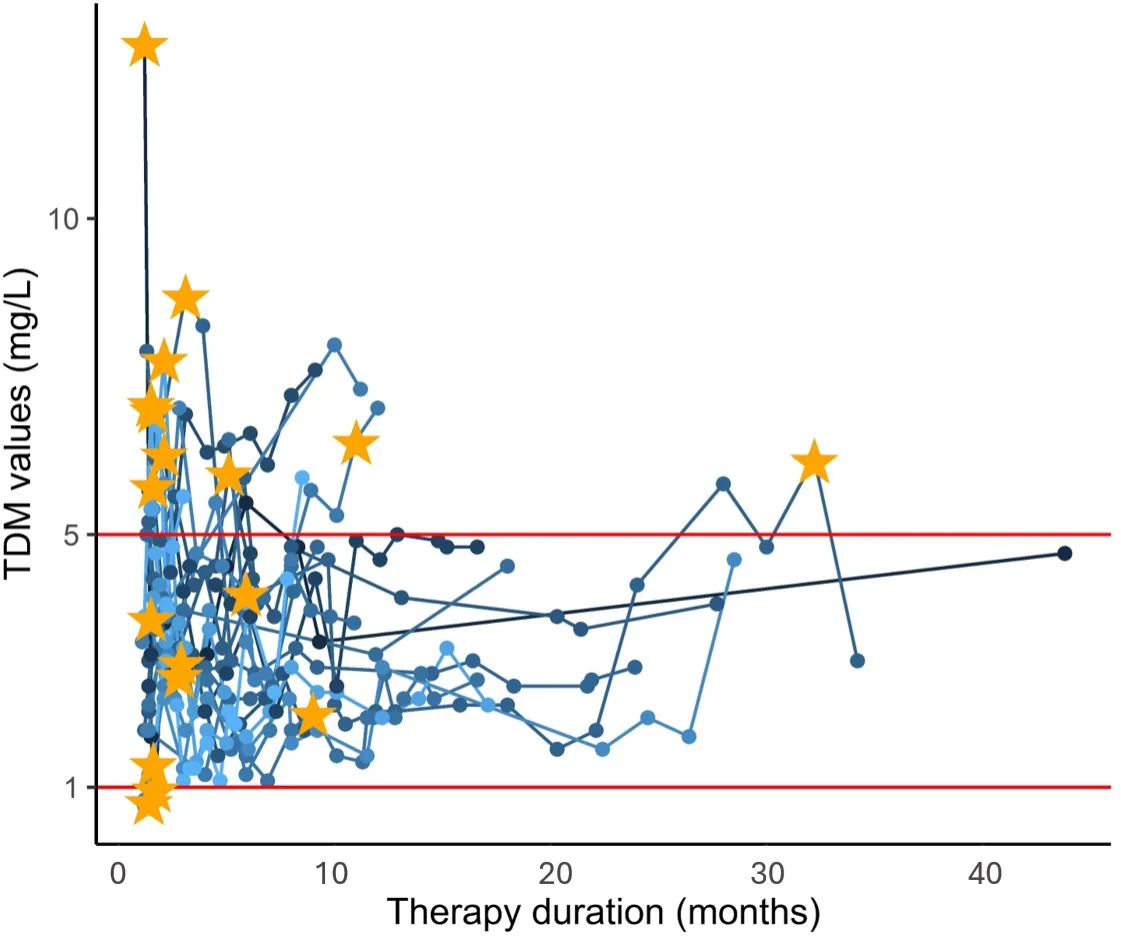
Longitudinal isavuconazole therapeutic drug monitoring (TDM) according to therapy duration for each patient. Orange stars indicate timepoints at which a posology adjustment was made. Red horizontal lines demarcate the therapeutic range (1–5 mg/L).

A total of 233 TDM measurements were recorded (mean 8.3 per patient). Most patients (67.9%, 19/28) maintained at least 80% of their values within the therapeutic range (1–5 mg/L); 17.6% (41/233) of individual measurements fell outside this range, predominantly supratherapeutic (38/41) (Fig. 3).

##### Dose adjustments and final regimen

Half of the patients (50.0%, 14/28) required at least one dose adjustment, after a median of 32.5 days from treatment initiation (IQR: 16–124); timing, indications, and outcomes of first and second adjustments are detailed in full in Table 3. Adjustments were most often prompted by supratherapeutic TDM levels, and complete resolution was achieved in all patients who required a second adjustment. Five patients (17.9%) died from causes unrelated to CPA during the treatment course. At the end of treatment or last follow-up, 57.1% of patients remained on the standard 200 mg daily dose, while 11/28 (39.3%) were maintained on a reduced dose of 100 mg daily, and one patient (3.6%) on 100 mg four times per week.

**TABLE 3:** Isavuconazole dose adjustments, clinical management, and final dosing regimen in patients receiving isavuconazole^*a*^

	Total patients receiving isavuconazole (*N* = 28), *n* (%)
First dose adjustment	
Patients requiring 1st adjustment	14 (50.0)
Time to first dose adjustment, days, median [IQR]	32.5 [16–124]
At 1st TDM	5 (35.7)
At 2nd TDM	3 (21.4)
At 3rd TDM or later	6 (42.9)
Reason for 1st adjustment	
Supratherapeutic TDM levels	10 (35.7)
Gastrointestinal symptoms	3 (10.7)
Liver toxicity (DILI)	3 (10.7)
Resolution after 1st adjustment	
Yes	11 (39.3)
No	3 (10.7)
Second dose adjustment	
Patients requiring 2nd adjustment	5 (17.9)
Reason for 2nd adjustment	
Subtherapeutic TDM levels	3 (10.7)
Supratherapeutic TDM levels	1 (3.6)
Symptomatic recurrence	1 (3.6)
Resolution after 2nd adjustment	5 (17.9)
Dose at end of treatment/last follow-up	
200 mg daily (standard dose)	16 (57.1)
100 mg daily (reduced dose)	11 (39.3)
100 mg four times per week	1 (3.6)

^*a*^ TDM, therapeutic drug monitoring; CPA, chronic pulmonary aspergillosis.

##### Cost estimations

Dose optimization through TDM-guided dose reduction resulted in a substantial decrease in drug costs. Based on the institutional acquisition cost of €34.8 per 100 mg capsule, the estimated total cost under a standard dosing regimen (200 mg daily throughout a median treatment duration of 10.6 months [IQR: 5.8–16.0]) would have been €715,001, corresponding to a median per-patient cost of €22,133 (IQR: €12,128–€33,373). The actual expenditure, accounting for TDM-guided dose reductions to 100 mg daily or 100 mg four times per week in eligible patients, was €558,331, with a median per-patient cost of €17,470 (IQR: €11,871–€29,049)—an estimated total saving of €156,670 (21.9%).

### Clinical outcomes

At the end of follow-up, treatment was completed in 25/40 patients (62.5%): 9 patients who received voriconazole throughout, and 16 who completed treatment on isavuconazole, either as first-line therapy or following a switch from voriconazole (including one patient who relapsed while on voriconazole and was subsequently switched to isavuconazole as a new independent treatment course, which was completed successfully). A total of 6/40 patients (15.0%) died during follow-up (1 on voriconazole, 5 on isavuconazole at the time of death; none of the deaths was attributed to CPA), while 4/40 patients (10.0%) discontinued treatment, either autonomously or in favor of a palliative approach (2 on voriconazole and 2 on isavuconazole before discontinuation).

Among the remaining patients, 5/40 (12.5%) patients, all on isavuconazole, completed at least 18 months of treatment but, given their underlying comorbidities, immune suppression, and good tolerability of isavuconazole, were maintained on isavuconazole as long-term suppressive therapy (Fig. 1).

## DISCUSSION

In our cohort, 70% of the total population ultimately received isavuconazole, either as an initial therapy or following a treatment switch. This frequent transition was primarily driven by the well-known poor tolerability and unpredictable pharmacokinetic properties of voriconazole; more than half of the patients who initiated voriconazole subsequently required a switch to isavuconazole due to failure to achieve therapeutic concentrations or TDM-confirmed toxicities (11). Of note, a switch from voriconazole to itraconazole would offer limited clinical advantage in this context, as these agents share similar pharmacokinetic variability and tolerability concerns, while posaconazole, despite its more favorable tolerability compared with itraconazole and voriconazole, remains subject to the same extensive cytochrome P450-mediated drug-drug interactions that represented the primary driver for isavuconazole use in our cohort (12). Isavuconazole, with its distinct safety and pharmacokinetic profile, represents a genuinely different therapeutic option for patients discontinuing voriconazole, as also acknowledged by the British Thoracic Society clinical statement, which includes isavuconazole as an alternative in cases of significant drug-drug interactions or intolerance to other azoles (13).

The extensive drug-drug interaction profile of voriconazole significantly limits its clinical utility, particularly in patients requiring antimycobacterial therapy. Rifamycin coadministration often precludes voriconazole due to profound cytochrome P450 induction, whereas isavuconazole offers a more manageable profile, with emerging evidence suggesting easier maintenance of therapeutic antifungal levels (14–16).

The higher mortality observed in the isavuconazole group should not be interpreted as a drug-related effect, nor as a meaningful comparison between treatments. Patients initiated on isavuconazole had a substantially greater burden of immunocompromising conditions, with active or recent malignancies present in 57.1% vs 23.1%, and the choice of isavuconazole as first-line therapy in these patients was itself driven by the need to avoid voriconazole-related toxicity in a particularly fragile population. This baseline imbalance, inherent to the retrospective and non-randomised design, precludes any meaningful comparison of mortality between the two treatment groups.

Data on isavuconazole in CPA patients remain scarce. A recent systematic review confirmed that only small studies are available, underscoring the need for further evidence (6). Bongomin et al. reported a significantly higher rate of adverse events in patients receiving voriconazole compared to isavuconazole (86% vs 60%, *P* = 0.02) in a retrospective cohort of 41 CPA patients (17). The safety profile of isavuconazole compared to voriconazole is further confirmed by the phase 3 trial by Kohno et al., in which adverse drug reactions were less frequent with isavuconazole than with voriconazole (61.5% vs 85.2%), with notably lower rates of hepatobiliary and ocular toxicity, consistent with the pattern observed in our cohort (18).

Beyond tolerability, no patient treated with isavuconazole in our cohort experienced a clinical relapse or required a switch due to treatment failure. The single relapse recorded in our cohort occurred in a patient receiving voriconazole. While our study was not designed to formally assess efficacy and the sample size precludes definitive conclusions, this observation is reassuring regarding long-term isavuconazole use in CPA.

Although isavuconazole was initially considered not to require routine TDM, owing to its predictable pharmacokinetic profile, a growing body of real-world evidence now demonstrates a clear relationship between serum concentrations and toxicity risk (5, 8, 19, 20). These findings support the rationale for early, proactive monitoring in long-term treatment settings such as CPA, where cumulative exposure over months may compound this risk.

In our cohort, TDM provided clinically actionable information: nearly 18% of isavuconazole TDM assessments were outside the therapeutic range, highlighting a non-negligible pharmacokinetic variability. Of these, 38 (92.7%) were supratherapeutic rather than subtherapeutic, indicating that pharmacokinetic variability in this population is predominantly driven by overexposure. Notably, the median initial isavuconazole trough concentration in our cohort was 5.1 mg/L (IQR: 3.3–6.2), at the upper boundary of the target range, suggesting that standard dosing (200 mg daily) may lead to supratherapeutic exposure in a substantial proportion of CPA patients from the outset. These observations reinforce the rationale for early, proactive TDM rather than reactive monitoring triggered by clinical toxicity. Our findings align with Kosmidis et al., who reported that a reduced dose of 100 mg daily can maintain adequate plasma concentrations (19). Interestingly, one patient in our cohort was maintained with a dose of 100 mg four times weekly, a regimen made pharmacokinetically feasible by isavuconazole’s long elimination half-life (~130 h), which allows extended dosing intervals while still maintaining steady-state trough concentrations (7).

Kosmidis et al. also showed that the mean isavuconazole level at first TDM was significantly higher in patients who developed adverse events compared to those who did not (5.5 vs 4.2 mg/L, *P* = 0.032), supporting the role of early TDM in identifying patients at risk of toxicity before clinical manifestations occur (19). While prospective validation is still needed, the available evidence (including our own findings) suggests that systematic TDM may facilitate early detection of supratherapeutic concentrations and contribute to a safer long-term use of isavuconazole.

The use of a more conservative upper threshold in our center (5 mg/L vs 6 mg/L in Kosmidis et al.) may further explain the favorable tolerability observed in our cohort. However, supratherapeutic concentrations in our patients were managed through TDM-guided dose reduction rather than treatment discontinuation (19). This underscores TDM not merely as a monitoring tool but as an active instrument for dose optimization and treatment preservation.

In our cohort, we observed a significant proportion of patients (39.3%) who maintained adequate therapeutic concentrations even after a reduction in isavuconazole posology; notably, this dose de-escalation led to a substantial decrease in drug-related costs. To date, cost-effectiveness studies for isavuconazole remain scarce and are predominantly focused on invasive aspergillosis, leaving a notable gap in the literature regarding CPA (21–23). To our knowledge, this is the first study to provide real-world insights into the drug acquisition cost savings associated with isavuconazole dose optimization in a chronic setting. While a formal cost-effectiveness analysis was not performed, our findings serve as a foundational evidence base for future research exploring the economic sustainability and potential advantages of isavuconazole for the long-term management of CPA.

This study has several limitations. The retrospective single-center design limits generalizability and introduces indication bias. The small sample size reduces statistical power for subgroup analyses. The uneven distribution of baseline characteristics (particularly higher malignancy burden in the isavuconazole group) limits direct comparability between groups. As no official TDM targets for isavuconazole have been defined to date by international guidelines, the 1–5 mg/L range adopted in our institution reflects real-world evidence rather than a validated consensus target (see Materials and Methods). Notably, the vast majority of out-of-range TDM measurements in our cohort were supratherapeutic (38/41, 92.7%), consistent with the exposure-toxicity relationship supporting the upper threshold adopted at our institution. Drug acquisition costs are, separately, inherently dependent on the specific center and healthcare market; nonetheless, the principle that TDM-guided dose reduction can generate meaningful cost savings is likely to generalize, even where the absolute figures reported here may not. Adverse events were captured only when they prompted a dose adjustment or switch rather than through systematic symptom screening; while this implies that unrecorded symptoms were, by definition, manageable without intervention, it also means that the true frequency and severity of adverse events in this cohort may be underestimated. The focus on voriconazole and isavuconazole limits the generalizability of findings to patients managed with other first-line triazoles. Finally, a small proportion of patients had not completed treatment at data extraction; all of these, however, were on long-term suppressive therapy and had already completed at least 12 months of antifungal treatment, limiting the impact on outcome completeness.

Strengths include the systematic use of TDM throughout treatment, providing one of the most detailed pharmacokinetic data sets for long-term isavuconazole use in CPA reported to date, and the real-world setting reflecting the complexity of patients encountered in routine practice. Finally, the inclusion of a cost analysis represents a novel contribution, providing a foundation for future cost-effectiveness research on isavuconazole in chronic treatment settings.

### Conclusions

In this real-world cohort of CPA patients, isavuconazole demonstrated a favorable tolerability profile compared to voriconazole, with no treatment discontinuations due to adverse events. The high rate of switch from voriconazole observed in our cohort, driven by toxicity, subtherapeutic drug levels, and drug-drug interactions, further underscores the limitations of current first-line therapy in complex, multimorbid patients. Systematic TDM, although not currently recommended by international guidelines, provided clinically actionable information, enabling early detection of supratherapeutic concentrations, timely dose adjustments, and safe long-term administration. A substantial proportion of patients maintained adequate plasma concentrations on a reduced dose of 100 mg daily, with significant implications for treatment costs. Our findings support isavuconazole as a viable, better-tolerated, and potentially more sustainable alternative for CPA management, and advocate for the prospective evaluation of routine TDM in this setting.

## Data Availability

The data set analyzed during the current study contains sensitive patient-level clinical information and is not publicly available, in accordance with Italian and EU data protection regulations (GDPR), and the terms of the Institutional Review Board approval described in the Ethics section. De-identified data are available from the corresponding author upon reasonable request, subject to institutional approval.

## References

[1] Denning DW. 2024. Global incidence and mortality of severe fungal disease. Lancet Infect Dis 24:e428–e438. doi:10.1016/S1473-3099(23)00692-8 38224705

[2] Denning DW, Cadranel J, Beigelman-Aubry C, Ader F, Chakrabarti A, Blot S, Ullmann AJ, Dimopoulos G, Lange C. 2016. Chronic pulmonary aspergillosis: rationale and clinical guidelines for diagnosis and management. Eur Respir J 47:45–68. doi:10.1183/13993003.00583-2015 26699723

[3] Sengupta A, Ray A, Upadhyay AD, Izumikawa K, Tashiro M, Kimura Y, Bongomin F, Su X, Maitre T, Cadranel J, de Oliveira VF, Iqbal N, Irfan M, Uzunhan Y, Aguilar-Company J, Munteanu O, Beardsley J, Furuuchi K, Takazono T, Ito A, Kosmidis C, Denning DW. 2025. Mortality in chronic pulmonary aspergillosis: a systematic review and individual patient data meta-analysis. Lancet Infect Dis 25:312–324. doi:10.1016/S1473-3099(24)00567-X 39617023

[4] Ullmann AJ, Aguado JM, Arikan-Akdagli S, Denning DW, Groll AH, Lagrou K, Lass-Flörl C, Lewis RE, Munoz P, Verweij PE, et al.. 2018. Diagnosis and management of aspergillus diseases: executive summary of the 2017 ESCMID-ECMM-ERS guideline. Clin Microbiol Infect 24 Suppl 1:e1–e38. doi:10.1016/j.cmi.2018.01.002 29544767

[5] Lewis JS, Wiederhold NP, Hakki M, Thompson GR. 2022. New perspectives on antimicrobial agents: isavuconazole. Antimicrob Agents Chemother 66:e0017722. doi:10.1128/aac.00177-22 PMC9487460 35969068

[6] de Oliveira VF, Taborda M, Katayose JT, Dos Santos Prates BV, de Souza Ritter IC, Gonçalves Kono Magri AS, Abdala E, Magri MMC. 2025. Isavuconazole in chronic pulmonary aspergillosis: what is the evidence? J Infect Chemother 31:102704. doi:10.1016/j.jiac.2025.102704 40220930

[7] McCreary EK, Davis MR, Narayanan N, Andes DR, Cattaneo D, Christian R, Lewis RE, Watt KM, Wiederhold NP, Johnson MD. 2023. Utility of triazole antifungal therapeutic drug monitoring: Insights from the society of infectious diseases pharmacists. Pharmacotherapy 43:1043–1050. doi:10.1002/phar.2850 37459118

[8] Furfaro E, Signori A, Di Grazia C, Dominietto A, Raiola AM, Aquino S, Ghiggi C, Ghiso A, Ungaro R, Angelucci E, Viscoli C, Mikulska M. 2019. Serial monitoring of isavuconazole blood levels during prolonged antifungal therapy. J Antimicrob Chemother 74:2341–2346. doi:10.1093/jac/dkz188 31119272

[9] Baccelli F, Campoli C, Colli M, Gatti M, Gottardi F, Pagano G, Venturelli F, Leardini D, Muratore E, Bossù G, Pea F, Viale P, Giannella M, Belotti T, Prete A, Masetti R. 2026. Isavuconazole as primary antifungal prophylaxis in pediatric hematological patients: a clinical and pharmacokinetic analysis. Antimicrob Agents Chemother 70:e0045126. doi:10.1128/aac.00451-26 PMC13435733 42360175

[10] Cusato J, Cafaro A, Cairoli S, Cangemi G, Cattaneo D, Di Paolo A, Menna P, Simeoli R, Tempestilli M, Goffredo BM. 2026. Indicazioni per l’armonizzazione degli intervalli terapeutici per i farmaci antifungini. Biochim Clin 50. doi:10.23736/S0393-0564.25.00088-3

[11] Li H, Lu Q, Xu B, Li A, Fang J, He X, et al.. 2026. Genetic and clinical predictors of voriconazole pharmacokinetics and hepatotoxicity: focused on CYP2C19 normal and intermediate metabolizers. J Antimicrob Chemother 81:dkag065. doi:10.1093/jac/dkag065 41766350

[12] Zhao YJ, Khoo AL, Tan G, Teng M, Tee C, Tan BH, Ong B, Lim BP, Chai LYA. 2016. Network meta-analysis and pharmacoeconomic evaluation of fluconazole, itraconazole, posaconazole, and voriconazole in invasive fungal infection prophylaxis. Antimicrob Agents Chemother 60:376–386. doi:10.1128/AAC.01985-15 PMC4704197 26525782

[13] Brown JS, Armstrong-James D, Ayling-Smith J, Backx M, Coleman M, Connell D, Dennison P, Downey DG, Lynch F, Lim WS, White J, Baxter C. 2025. British thoracic society clinical statement on aspergillus-related chronic lung disease. Thorax 80:3–21. doi:10.1136/thorax-2024-222567 40404294

[14] Sehgal IS, Soundappan K, Agarwal R, Muthu V, Dhooria S, Prasad KT, Salzer HJF, Cornely OA, Aggarwal AN, Chakrabarti A. 2025. Prevalence of chronic pulmonary aspergillosis in patients with mycobacterial and non-mycobacterial tuberculosis infection of the lung: a systematic review and meta-analysis. Mycoses 68:e70060. doi:10.1111/myc.70060 40265658

[15] Li T-Y, Liu W, Chen K, Liang S-Y, Liu F. 2017. The influence of combination use of CYP450 inducers on the pharmacokinetics of voriconazole: a systematic review. J Clin Pharm Ther 42:135–146. doi:10.1111/jcpt.12493 28177134

[16] Shah S, Clarke L, Lee T, Georgiades L, Smith BJ, Venkataramanan R, Rivosecchi RM. 2026. Pharmacokinetic interaction between isavuconazole and rifabutin in a real-world setting. Mycoses 69:e70157. doi:10.1111/myc.70157 PMC12828710 41574599

[17] Bongomin F, Maguire N, Moore CB, Felton T, Rautemaa-Richardson R. 2019. Isavuconazole and voriconazole for the treatment of chronic pulmonary aspergillosis: a retrospective comparison of rates of adverse events. Mycoses 62:217–222. doi:10.1111/myc.12885 30570179

[18] Kohno S, Izumikawa K, Takazono T, Miyazaki T, Yoshida M, Kamei K, Ogawa K, Taniguchi S, Akashi K, Tateda K, Mukae H, Miyazaki Y, Okada F, Kanda Y, Kakeya H, Suzuki J, Kimura S-I, Kishida M, Matsuda M, Niki Y. 2023. Efficacy and safety of isavuconazole against deep-seated mycoses: a phase 3, randomized, open-label study in Japan. J Infect Chemother 29:163–170. doi:10.1016/j.jiac.2022.10.010 36307059

[19] Kosmidis C, Otu A, Moore CB, Richardson MD, Rautemaa-Richardson R. 2020. Isavuconazole therapeutic drug monitoring during long-term treatment for chronic pulmonary aspergillosis. Antimicrob Agents Chemother 65:01511–01520. doi:10.1128/AAC.01511-20 PMC7927812 33077653

[20] Huang E, Wittenberg R, Dray JV, Fine J, Robison E, Wilson M, Trigg K, Bays DJ, Chee M, Thompson GR 3rd. 2025. Isavuconazole therapeutic drug monitoring and association with adverse events. J Antimicrob Chemother 80:1702–1706. doi:10.1093/jac/dkaf128 PMC12129579 40289250

[21] Engelhard M, Wingen-Heimann SM, Grüner B, Vehreschild M, Kessel J, Ehrlich S, Spiekermann K, Schalk E, Baermann B-N, Vehreschild JJ, Pütz SM. 2026. Clinical and health economic impact of isavuconazole for treatment of invasive aspergillosis and mucormycosis: a retrospective, matched multicentre cohort study in Germany. Infection 54:377–387. doi:10.1007/s15010-025-02674-x PMC12864223 41171517

[22] Azanza JR, Grau S, Vázquez L, Rebollo P, Peral C, López-Ibáñez de Aldecoa A, López-Gómez V. 2021. The cost-effectiveness of isavuconazole compared to voriconazole, the standard of care in the treatment of patients with invasive mould diseases, prior to differential pathogen diagnosis in Spain. Mycoses 64:66–77. doi:10.1111/myc.13189 PMC7894146 32989796

[23] Araujo GLV, Murta Amaral L, Ponzio V, Rocha JL. 2024. Economic and budgetary impact evaluation of isavuconazole (Cresemba) versus voriconazole (Vfend) for the treatment of patients with possible invasive aspergillosis from the perspective of the Brazilian supplementary health system. PLoS One 19:e0299056. doi:10.1371/journal.pone.0299056 PMC10906891 38427635

